# Unravelling complex relationships between health literacy and shared decision-making: a cross-sectional study in patients attending rheumatology rehabilitation

**DOI:** 10.1016/j.ero.2026.02.014

**Published:** 2026-03-05

**Authors:** Lisbeth Skovly Nielsen, Kirsten Lykke Knak, Brian Clausen, Kamila Adellund Holt, Sofie Ronja Petersen, Jette Primdahl, Ann Bremander

**Affiliations:** 1 The Danish Centre for Expertise in Rheumatology, Danish Hospital for Rheumatic Diseases, University Hospital of Southern Denmark, Sønderborg, Denmark; 2 Department of Regional Health Research, University of Southern Denmark, Odense, Denmark; 3 The Danish Rheumatism Association’s Rehabilitation Centre, Sano, Skælskør*,* Denmark; 4 Department of Clinical Research, University Hospital of Southern Denmark, Aabenraa, Denmark; 5 Section of Rheumatology, Department of Clinical Sciences, Lund University, Lund, Sweden; 6 Spenshult Research and Development Centre, Halmstad, Sweden

## Abstract

**Objectives:**

Consideration of health literacy (HL) in shared decision-making (SDM) is key to improving rehabilitation outcomes and addressing social health inequities. Therefore, the aim of this study was to investigate the relationship between HL skills and patient-perceived involvement in SDM within goal setting in rheumatology rehabilitation.

**Methods:**

A multicentre online survey was sent to patients attending goal setting in rehabilitation. SDM was measured with the patient-reported questionnaire CollaboRATE (CollaboRATE is a brief patient‑reported measure of shared decision making; the top score reflects optimal SDM) and HL skills with the Health Literacy Questionnaire (HLQ, 9 scales). Associations between HLQ scores and CollaboRATE (suboptimal vs optimal [top score]) were investigated by polynomial logistic regression analyses adjusted for possible covariates.

**Results:**

A total of 408/514 (79%) patients responded to the survey; 401 (78%) completed both questionnaires (mean age 58.5 years [SD: 12.4], disease duration 11.4 years [SD: 11.2], 85% women). A linear relationship between HLQ and CollaboRATE was demonstrated in 2/9 HLQ scales, while 5/9 HLQ scales showed a U/J-shaped pattern (*P* < .039). Higher HLQ scores were associated with the greatest probability of reporting optimal SDM, followed by lower scores. In contrast, moderate scores were more frequently associated with suboptimal SDM. Two HLQ scales showed no association with SDM. More men than women reported suboptimal SDM (64% vs 48%, *P* = .02).

**Conclusions:**

This study found a complex relationship between HL skills and patients’ experience of involvement in SDM, and these findings highlight the need for tailored support from healthcare providers to facilitate meaningful SDM, particularly for those facing challenges related to HL including individuals with moderate HL skills.

**WHAT IS ALREADY KNOWN ON THIS TOPIC**

• Involving patients in shared decision-making (SDM) is a core component in rehabilitation and goal setting, supporting person-centred care.
• Active participation in healthcare decisions depends on the person’s ability to seek, understand, evaluate, and apply health information—core skills of health literacy (HL), which is increasingly recognised as a key determinant of health.
• Although research on SDM and HL has developed in parallel, and their required skills overlap meaningfully, the relationship between SDM and specific HL dimensions has not yet been thoroughly explored within the population with rheumatic and musculoskeletal disease.

**WHAT THIS STUDY ADDS**

• This study offers new insights into how different dimensions of HL relate to patients’ experience of SDM within goal setting in rheumatology rehabilitation.
• Moderate HL skills in 1 or more dimensions may be associated with feeling less involved in SDM—and even less involved than those with lower HL skills, suggesting that support needs can be nuanced and are not always easily identified. This challenges the commonly held assumption of a consistent, linear relationship between HL and SDM.
• The findings also point to possible sex-related differences, with men more often reporting suboptimal SDM, indicating the need for further attention to gender aspects of patient engagement in SDM.

**HOW THIS STUDY MIGHT AFFECT RESEARCH, PRACTICE OR POLICY**

• These findings may encourage clinicians to reflect more deeply on the varied HL profiles of their patients and consider how tailored communication can support meaningful SDM.
• Patients with moderate HL skills in 1 or more dimensions might benefit from more personalised guidance.
• Future research should explore how organisations and healthcare providers can adapt their approaches to meet diverse HL needs. More research on associations between HL dimensions and SDM is warranted, helping to refine both measurement and practice.

Alt-text: Unlabelled box dummy alt text

## INTRODUCTION

A goal-directed approach to achieve behaviour change is crucial in rehabilitation, where goal setting is considered the best practice and patient involvement in decision-making is central [1]. Studies suggest that patient involvement in goal setting improves adherence to rehabilitation and increases motivation to achieve goals [1,2]. Despite the importance of patient involvement in goal setting, implementing it in rehabilitation is challenging, and various strategies are available [3]. Shared decision-making (SDM) is a commonly used strategy in which patients collaborate with healthcare providers (HPs) to decide on the best course of action during the medical encounter [4], and SDM has also proven useful in strengthening the goal-setting process [2]. Grounded in patient autonomy and informed consent, SDM is recognised for its potential to improve patients’ clinical outcomes through structured information exchange and alignment of preferences [5]. Strategies to involve patients in the goal-setting process, such as SDM, are known to improve satisfaction with treatment decisions, ownership of goals, and functioning [1,2] and are recommended by the European Alliance of Associations for Rheumatology (EULAR) [6]. By focusing on patients’ preferences, SDM has the potential to enhance patients’ sense of being heard and strengthen trust in HPs [7]. However, evidence shows that patients often feel less involved than desired [8], and some patients with rheumatic and musculoskeletal diseases (RMDs) involved in rheumatology rehabilitation programmes struggle to manage the responsibility required during SDM [9].

Positive outcomes of SDM depend on factors such as having sufficient time for discussion, open and trusting communication, and a supportive environment [10]. In addition to factors related to the HPs and the organisation, patient-related factors such as the ability to seek, understand, critically assess, and act upon health information, known as health literacy (HL) skills [11], also play a crucial role [12]. HL encompasses the ability to read and understand health information, navigate the healthcare system, communicate and engage with HPs, understand planned treatments, and be aware of one’s rights within the healthcare system [13]. Recognised by the World Health Organization (WHO) as a key health determinant [14], low HL skills are associated with financial deprivation and low educational levels [[15], [16], [17]], poorer self-assessed health [18], underutilisation of preventive healthcare services [11], diminished physical and mental health [19], and multimorbidity [17,18]. Despite its importance, HL is often overlooked, though organisations increasingly acknowledge the need for approaches and communication strategies that address diverse HL challenges [20]. HL is essential for engaging in SDM [5,12], as insufficient HL correlates with poor treatment adherence and difficulties in managing chronic conditions [21]. Surveys indicate that insufficient HL affects about 50% of Europeans [17] and 40% of the Danish population [22].

In rheumatology, HL is increasingly recognised by HPs [23], as low HL has been linked to higher disease activity, reduced medication adherence, mental health challenges, and poorer functional outcomes among patients with inflammatory arthritis [23]. Since HL skills are modifiable [11], assessing them is essential for enabling active engagement in SDM [12]. HL skills align closely with those required for SDM, and Muscat et al [12] expanded the ‘model of SDM’ [24] by integrating HL skills to enhance patient engagement in healthcare interactions. If interventions such as interdisciplinary rehabilitation fail to address specific HL needs, vulnerable patients risk marginalisation, exacerbating social health inequalities [25]. The relationship between HL and SDM in patients with RMDs remains underexplored, despite its relevance for improving rehabilitation outcomes and reducing social disparities. Therefore, this study aimed to examine the association between HL and patient-perceived involvement in SDM during goal setting in an interdisciplinary rehabilitation setting.

## METHODS

### Study design

A cross-sectional, multicentre questionnaire study was conducted.

### Setting and participants

Data were collected at 2 Danish rehabilitation centres for adult patients with RMDs, who face complex biopsychosocial challenges such as fatigue, cognitive impairment, pain, and functional limitations due to their condition. Centre 1 is a rehabilitation centre where patients are expected to be self-reliant, whereas centre 2 is a hospital that admits patients with multiple comorbidities and those requiring around-the-clock care. At both centres, rehabilitation goals are collaboratively set by the patient and an interdisciplinary team of HPs, including physiotherapists, occupational therapists, nurses, rheumatologists, and possibly social workers or psychologists. The goals are individually tailored to patients’ biopsychosocial challenges, adjusted as needed during the intervention period, and evaluated upon discharge. Patients admitted for team rehabilitation at 1 of the 2 centres were consecutively invited and enrolled in the study between April 2021 and June 2022.

### Data collection

During the initial consultation before their upcoming rehabilitation stay, HPs informed the patients about the study. Patients who provided written consent received the questionnaires after the goal-setting conversation, either in their private digital mailbox or on paper, depending on their preference. At both centres, tablets were available with access to the digital version of the questionnaires, and staff members were present to assist if necessary. Participants were given 1 week to complete the questionnaires, with a reminder sent on the fifth day if no response had been received.

The survey included patient-reported questionnaires assessing SDM, HL, health-related quality of life, psychological well-being, and demographic variables. All selected instruments were deemed appropriate for the study population based on their psychometric properties and established use in similar clinical and research contexts. The survey was pilot tested by 2 patient research partners and 2 HPs to ensure feasibility, and their input led to minor rephrasing of the introductory text of the survey. The participants were explicitly instructed to reflect on the goal-setting conversation when responding to items related to perceived involvement. The questionnaires were administered electronically using Research Electronic Data Capture (REDCap), a secure, web-based application designed to support data collection for research studies.

#### SDM

The participants’ perception of being informed and involved in SDM within goal setting in rehabilitation was measured by the Danish version of CollaboRATE [26]. CollaboRATE is a short, generic questionnaire assessing the extent to which 3 core tasks in SDM are present in an encounter between the patient and HPs: (i) the patient’s perception of how well their health issue was explained, (ii) how much the HP listened to the patient’s preferences, and (iii) how well the patient’s preferences were included in decisions about what to do next [26,27]. Each of the 3 questions has a 10-point scale, ranging from 0 (no effort) to 9 (a great deal of effort). The higher the score, the higher the perception of SDM. CollaboRATE offers 2 scoring methods: a mean score and a top score. CollaboRATE is subject to a ceiling effect; thus, we chose the top score as it discriminates more effectively between the absence (no) and presence (yes) of any level of SDM [26]. CollaboRATE has demonstrated good psychometric properties, including strong concurrent validity and acceptable intrarater reliability [26].

#### HL

Participants’ HL skills were measured by the Danish version of the Health Literacy Questionnaire (HLQ) [13]. The HLQ consists of 44 items covering 9 conceptually distinct dimensions (scales) of HL (Table 1). An average score is calculated for each scale; the higher the score, the higher HL skills on that specific scale. Response options for each scale vary from a 4-point Likert scale (1-4: strongly disagree, disagree, agree, and strongly agree) to a 5-point Likert scale (1-5: cannot do or always difficult, usually difficult, sometimes difficult, usually easy, and always easy). The Danish validation demonstrates good internal consistency across the scales and supports a robust 9-factor confirmatory structure [13]. According to the development paper by Osborne et al [28], scales 1 and 2 (*Feeling understood and supported by healthcare providers* and *Having sufficient information to manage my health*)—are particularly useful for informing decisions at the organisational level. Scales 3, 4, and 5 (*Actively managing my health, Having social support for health,* and *Critical appraisal of health information*) are more relevant for guiding decisions related to individual-level outcomes, whereas scales 6, 7, and 9 (*Ability to actively engage with healthcare providers, Navigating the healthcare system,* and *Understanding health information well enough to know what to do*) provide insights applicable to both organisational and individual needs [28].

**Table 1:** Description of Health Literacy Questionnaire parts 1 and 2, its scales, and number of items

HLQ scale	Description
Part 1^a^	
1	Feeling understood and supported by healthcare providers (4 items)
2	Having sufficient information to manage my health (4 items)
3	Actively managing my health (5 items)
4	Having social support for health (5 items)
5	Critical appraisal of health information (5 items)
Part 2^b^	
6	Ability to actively engage with healthcare providers (5 items)
7	Navigating the healthcare system (6 items)
8	Ability to find good health information (5 items)
9	Understanding health information well enough to know what to do (5 items)

HLQ, Health Literacy Questionnaire.

a Agreement is measured on a 4-point Likert scale: (1) strongly disagree, (2) disagree, (3) agree, and (4) strongly agree.

b Experienced difficulty is measured on a 5-point Likert scale: (1) cannot do, or always difficult, (2) usually difficult, (3) sometimes easy, (4) usually easy, and (5) always easy.

Due to the complexity and ongoing development of the HL concept, there is currently no consensus on the most appropriate terminology for describing a person’s HL skills [29]. Therefore, we refer to ‘lower, moderate, and higher HLQ scores’ when presenting data from this study, and to ‘lower, moderate, and higher HL skills’ in the discussion, acknowledging that we are unable to define these levels or cut-offs with greater precision, as individuals may possess high HL skills in some dimensions while exhibiting lower skills in others [25].

#### Health-related quality of life

Health-related quality of life was assessed using the European Quality of Life, 5 dimensions, 5 levels (EQ-5D-5L) questionnaire [30]. The resulting index calculated by the Danish value set ranges from −0.76 (a state worse than death) to 1 (full health), reflecting individuals’ self-perceived health-related quality of life [31]. The Danish value set is grounded in a national study, making it appropriate for this population [31]. The EQ Visual Analogue Scale (VAS) for overall self-assessed health (EQ-5D-5L VAS) with scores ranging from 0 (worst) to 100 (best), was also applied [30].

#### Psychological well-being

Psychological well-being was assessed by the Hopkins Symptoms Checklist-5 (HSCL-5), a short form derived from a 25-item symptom checklist, identifying signs of anxiety and depression. The HSCL-5 comprises 5 items, each scored from best to worst, with values ranging from 1 (not at all) to 4 (extremely), and a reference period of the 2 preceding weeks [32]. Nordic validation has demonstrated good internal consistency and acceptable validity [32].

#### Demographic variables

Demographic variables included age, sex, primary RMD diagnosis, disease duration (years), number of comorbidities (0-1, 2-3, or >3), educational level (low: ≤11 years, medium: >11-14 years, or high: >14 years), previous rehabilitation interventions (yes/no, if ‘yes’ respondents were asked to specify the year), and whether the survey was completed digitally or on paper.

### Statistical analyses

We applied the CollaboRATE top score and dichotomised the respondents as follows: 1 (yes, optimal SDM) if they scored 9 on all 3 questions and 0 (no, suboptimal SDM) if they did not [26]. The EQ-5D-5L index was calculated based on the Danish EQ-5D-5L value set [31], and EQ-5D-5L VAS was described on the group level as mean and SD. An average score of >2 on the HSCL-5 indicates a high degree of mental health problems and defines the cut-off [32].

Associations between the CollaboRATE top score (dependent variable) and each of the 9 HLQ scales (independent variables) showed a nonlinear pattern and were therefore examined using polynomial logistic regression analyses, controlling for relevant explanatory variables. To select covariates, and thereby possible confounders for a multivariate analysis, a directed acyclic graph was created to get a visual presentation of the relationships and dependencies between the dependent and the independent variables (age, sex, diagnosis, disease duration, comorbidities, educational level, previous rehabilitation interventions, mode of administration of survey, HSCL-5, EQ-5D-5L index, and EQ-5D-5L VAS scores). Furthermore, we used the Akaike’s information criterion for model specification [33]. Results are presented graphically on a probability scale using margins plots derived from the logistic regression models. Only complete responses for CollaboRATE were analysed. Missing data from the independent variables were accounted for by the maximum likelihood approach in the primary model. A heterogeneity adjustment of the data from the 2 different centres was found to be negligible, as generalised estimating equations and ordinary logistic regression yielded nearly identical point and variance estimates. To account for intercorrelation between the 9 HLQ scales, each scale was included in a separate model, and model diagnostics of the logistic regression analyses were performed using partial residual plots [34].

An experienced statistician (SRP) was involved in all analyses. All statistical analyses were performed using Stata (version 18, StataCorp LLC) and the R-package *eq5d* (available at: https://www.R-project.org).

### Ethics

The study complied with the Helsinki Declaration [35] and the ethical guidelines of the Danish National Committee on Health Research Ethics [36]. In accordance with Danish law, survey studies do not require formal approval from regional ethics committees (case no: 20202000-117). All participants gave verbal and written informed consent, were assured of confidentiality, and could withdraw at any time before data analysis. To meet Danish data protection regulations and General Data Protection Regulation, data were securely stored and analysed within the OPEN (Open Patient data Explorative Network) platform, hosted by the Region of Southern Denmark.

### Patient research partners

Two patient research partners were actively involved to help ensure the clinical relevance of the study for individuals living with RMDs, which aligns with EULAR’s recommendations for the inclusion of patient representatives in rheumatology research [37]. They reviewed the introductory text, demographic items, and survey flow in both REDCap and the paper format to ensure clarity and feasibility.

## RESULTS

Of approximately 700 invited patients, 514 consented to participate and received the survey. Among these, 408 (79%) responded and 401 (78%) of these respondents fully completed both the HLQ and the CollaboRATE questionnaire. The nonresponders (n = 106, 21%) had a mean age of 58 years (SD = 14.4), and the majority (79%) were women. The responding participants’ mean age was 58.5 years (SD = 12.4), with a predominant female representation (85%). Among the participants, 35% had inflammatory arthritis, and 28% osteoarthritis, whereas chronic widespread pain accounted for 17%. A total of 33% reported more than 3 comorbidities. There were 16% of participants with less than 14 years of education. In total, 33% had prior experience with rehabilitation (Table 2). The 2 centres displayed some differences: centre 2 had a greater proportion of participants who opted for a paper-based rather than a digital survey (34% vs 20%, respectively), as well as a lower prevalence of participants with widespread pain/fibromyalgia than centre 1 (7% vs 29%, respectively). Suboptimal SDM communication was reported by 50% of the participants, with a larger proportion of men compared with women (64% vs 48%, *P* = .02). Descriptive data showed no other clinically relevant differences between participants who reported optimal vs suboptimal SDM (Table 3).

**Table 2:** Participant characteristics by sex

Characteristics	N	All	Sex	
			Women N = 347	Men N = 61
Age (y), mean (SD)	408	58.5 (12.4)	57.8 (12.4)	62.2 (12.0)
Primary diagnosis, n (%)	398			
Inflammatory arthritis		140 (35)	115 (34)	25 (42)
Osteoarthritis		113 (28)	98 (29)	15 (25)
Chronic widespread pain		66 (17)	64 (19)	2 (3)
Other RMD		79 (20)	61 (18)	18 (30)
Disease duration (y), mean (SD)	385	11.4 (11.2)	11.1 (10.8)	12.9 (13.4)
Comorbidities, n (%)	408			
0-1		112 (27)	90 (26)	22 (36)
2-3		162 (40)	139 (40)	23 (38)
≥4		134 (33)	118 (34)	16 (26)
Previous rehabilitation experience, n (%)	408	136 (33)	115 (33)	21 (34)
Educational level, n (%)	407			
1-11 y		19 (5)	13 (4)	6 (10)
> 11-14 y		45 (11)	34 (10)	11 (18)
>14 y		343 (84)	299 (86)	44 (72)
CollaboRATE (0/1),^a^ n (%)	401	201 (50)/200 (50)	163 (48)/179 (52)	38 (64)/21 (36)
HLQ^b^ mean (SD)				
1: Feeling understood and supported by healthcare providers	404	3.0 (0.6)	3.0 (0.6)	3.1 (0.5)
2: Having sufficient information to manage my health	404	2.7 (0.6)	2.7 (0.6)	2.8 (0.6)
3: Actively managing my health	404	2.8 (0.5)	2.8 (0.5)	2.9 (0.4)
4: Having social support for health	404	3.0 (0.6)	3.0 (0.6)	2.9 (0.5)
5: Critical appraisal of health information	404	2.7 (0.5)	2.8 (0.5)	2.7 (0.5)
6: Ability to actively engage with healthcare providers	403	3.4 (0.8)	3.3 (0.8)	3.6 (0.7)
7: Navigating the healthcare system	403	3.0 (0.7)	2.9 (0.7)	3.1 (0.7)
8: Ability to find good health information	403	3.5 (0.7)	3.5 (0.7)	3.4 (0.6)
9: Understand health information well enough to know what to do	403	3.6 (0.6)	3.6 (0.7)	3.5 (0.6)
HSCL-5 (0/1),^c^ n (%)	408	324 (79)/84 (21)	163 (48)/179 (52)	49 (80)/12 (20)
EQ-5D-5L index (0-1, worst-best), mean (SD)	402	0.6 (0.2)	0.6 (0.2)	0.6 (0.2)
EQ-5D-5L VAS (0-100, worst-best), mean (SD)	402	53.6 (17.7)	53.3 (17.3)	55.2 (20.1)

CollaboRATE, CollaboRATE is a brief patient‑reported measure of shared decision making; the top score reflects optimal SDM; EQ-5D-5L, European Quality of Life, 5 dimensions, 5 levels; HLQ, Health Literacy Questionnaire; HSCL-5, Hopkins Symptoms Checklist-5; RMD, rheumatic and musculoskeletal disease; VAS, Visual Analogue Scale.

a CollaboRATE 0 represents suboptimal shared decision-making, 1 represents optimal shared decision-making.

b Health Literacy Questionnaire, scales 1-5 scored from 1 to 4 (worst- best) and scales 6-9 scored from 1 to 5 (worst-best).

c Hopkins Symptoms Checklist, an average score on the HSCL-5 >2 indicates a high level of mental health problems (1) and was used as a cut off, and 0 represents no problems.

**Table 3:** Participant and HL characteristics by suboptimal vs optimal SDM measured by CollaboRATE

Characteristics	All N = 401	SDM	
		Suboptimal N = 201	Optimal N = 200
Age (y), mean (SD)	58.5 (12.4)	58.4 (12.2)	58.6 (12.7)
Sex, women n (%)	342 (85)	163 (81)	179 (90)
Previous rehabilitation experience, n (%)	135 (34)	64 (32)	71 (36)
Years since last rehabilitation, mean (SD)	6.4 (6.8)	6.2 (5.0)	6.5 (8.2)
Educational level, n (%)			
≤11 y	19 (5)	11 (5)	8 (4)
>11-14 y	42 (10)	22 (11)	20 (10)
>14 y	340 (85)	168 (84)	172 (86)
HLQ^a^ mean (SD)			
1: Feeling understood and supported by healthcare providers	3.0 (0.6)	2.9 (0.5)	3.2 (0.6)
2: Having sufficient information to manage my health	2.7 (0.6)	2.6 (0.5)	2.8 (0.6)
3: Actively managing my health	2.8 (0.5)	2.8 (0.5)	2.9 (0.5)
4: Having social support for health	3.0 (0.6)	2.9 (0.5)	3.0 (0.6)
5: Critical appraisal of health information	2.7 (0.5)	2.7 (0.5)	2.7 (0.6)
6: Ability to actively engage with healthcare providers	3.4 (0.8)	3.3 (0.7)	3.5 (0.8)
7: Navigating the healthcare system	3.0 (0.7)	2.9 (0.7)	3.1 (0.7)
8: Ability to find good health information	3.5 (0.7)	3.4 (0.7)	3.5 (0.7)
9: Understand health information well enough to know what to do	3.6 (0.6)	3.5 (0.7)	3.6 (0.6)
HSCL-5^b^, score >2, n (%)	78 (19)	42 (21)	36 (18)
EQ-5D index (0-1, worst-best), mean (SD)	0.65 (0.24)	0.64 (0.24)	0.65 (0.25)
EQ VAS score (0-100, worst-best), mean (SD)	53.6 (17.7)	51.8 (17.7)	55.4 (17.7)

CollaboRATE, CollaboRATE is a brief patient‑reported measure of shared decision making; the top score reflects optimal SDM; EQ-5D-5L, European Quality of Life, 5 dimensions, 5 levels; HLQ, Health Literacy Questionnaire; HSCL-5, Hopkins Symptoms Checklist-5; SDM, shared decision-making; VAS, Visual Analogue Scale.

a Health Literacy Questionnaire, scales 1-5 scored from 1 to 4 (worst-best) and scales 6-9 scored from 1 to 5 (worst-best).

b Hopkins Symptoms Checklist, an average score on the HSCL-5 > 2 indicates a high level of mental health problems (1) and was used as a cut off, and 0 represents no problems.

### The relationship between HL skills and perceived involvement in SDM

The results from the regression analyses are presented as marginal probabilities (Fig) and revealed linear associations between 2 of 9 HLQ scales, *Navigating the health system* (scale 7) and *Finding health information* (scale 8) and SDM, with a consistently positive relation.

**Figure:**
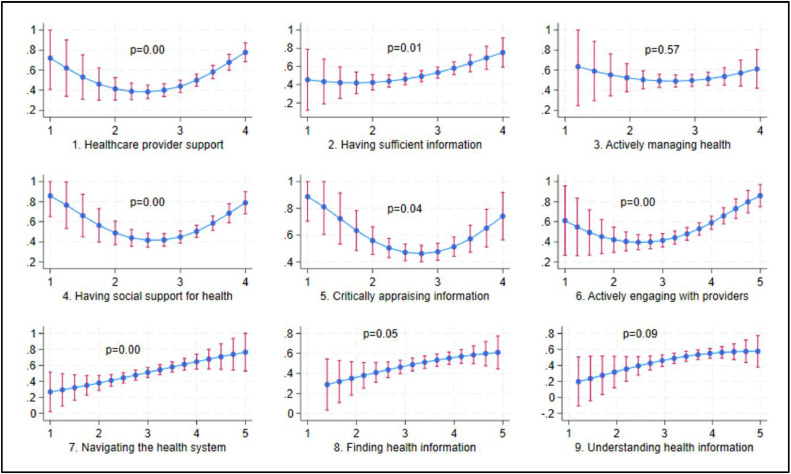
Marginal probabilities of reporting optimal SDM across the 9 HLQ scales. X-axis: HLQ scale mean values. Y-axis: marginal probabilities reflecting the predicted likelihood of reporting optimal SDM (CollaboRATE top score) for corresponding HLQ scores, with 95% CIs. CollaboRATE, (CollaboRATE is a brief patient‑reported measure of shared decision making; the top score reflects optimal SDM); HLQ, Health Literacy Questionnaire; SDM, shared decision-making.

A U-shaped or J-shaped pattern was seen for 5 of 9 HLQ scales: *Healthcare provider support* (scale 1), *Having sufficient information* (scale 2), *Having social support for health* (scale 4), *Critically appraising information* (scale 5), and *Actively engaging with providers* (scale 6). The associations were highly statistically significant for HLQ scales 1, 2, 4, and 6 (*P* ≤ .01) and significant for scale 5 (*P* = .039). Higher HLQ scores were associated with the greatest probability of reporting optimal SDM (CollaboRATE top score), followed by lower scores. In contrast, moderate HLQ scores were more frequently associated with suboptimal SDM. Two scales, *Actively managing health* (scale 3) and *Understanding health information* (scale 9), did not show any significant associations with SDM (Fig).

## DISCUSSION

The aim of this study was to examine the relationship between HL and patient-perceived involvement in SDM during goal setting in rheumatology rehabilitation, revealing a complex relationship. Both linear and nonlinear associations emerged, partly contrasting prior findings that suggest a linear relationship between these skills [38].

Linear associations were observed between HL skills and perceived SDM in 2 of the 9 HLQ scales: *Navigating the health system* and *Finding health information*. This indicates that as patients improve in accessing services, locating and understanding health information, their capacity for active participation in decision-making is likely to increase in a predictable, stepwise manner, consistent with the WHO’s definition of HL [39]. Five HLQ scales showed a nonlinear pattern with SDM, with both low and high scores linked to optimal SDM: *Healthcare provider support, Having sufficient information, Having social support for health, Critically appraising information,* and *Actively engaging with providers*. Interestingly, moderate HLQ scores on these scales were associated with suboptimal levels of SDM, contrasting prior research suggesting a linear relationship [38]. A plausible explanation is that individuals with moderate HL skills may have sufficient knowledge for HPs to expect active involvement, yet lack the confidence, communication skills, or contextual support needed to fully engage [10].

This mismatch between expectations and actual experience can lead to frustration and disengagement, resulting in lower perceived SDM. In addition, HPs may overestimate the capabilities of patients with moderate HL skills and provide less tailored support. In line with our findings, prior studies have indicated that individuals with higher HL skills tend to participate more actively in healthcare decision-making [12]. Those with lower HL skills may struggle to assess their needs [38], yet still report optimal SDM, likely due to a preference for a passive role within systems that do not explicitly invite patient participation in decision-making [40]. In a previous interview study in the same population, we found that some patients were quite satisfied when the HPs took full responsibility for setting the rehabilitation goals, as these patients acknowledged the HPs as experts [9]. These findings underscore the need for interventions that address diverse HL challenges and support relational and motivational aspects of SDM—not only for those with low HL but also for patients with moderate HL skills, who may be at risk of being overlooked, as shown in this study.

Half of the participants reported suboptimal SDM, which is concerning, as suboptimal SDM has been linked to reduced satisfaction and ownership of treatment goals [41], poorer rehabilitation outcomes [5], and a lower sense of being heard, which may diminish trust in HPs [7]. Our findings are in contrast to a British study, where only a third of the participants diagnosed with rheumatoid arthritis (RA) reported suboptimal SDM [42], likely reflecting differences in sample characteristics, outcome measures, settings, and study designs. In our study, men were more likely than women to report suboptimal SDM (64% vs 48%). Sex-related influences on health behaviours and outcomes remain understudied [43], underscoring the need for gender-responsive, person-centred care [44]. These findings should be interpreted cautiously due to the limited number of male participants and educational differences between the sexes.

Although our study focused on individuals with RMDs, the HLQ scores were comparable to Danish population studies [15,45] and a Dutch cohort involving patients with RA [25], suggesting that HL is a broader person-level attribute [46]. Although individual HL skills are central to SDM, organisational HL (OHL) also shapes patients’ ability to engage meaningfully in healthcare encounters [47]. OHL reflects how well healthcare organisations enable individuals to navigate, understand, and use information for informed decisions [47]. Given the complexity of person-centred goal setting, HPs must recognise the diverse HL challenges patients face and respond with appropriate support. The HL and Equity for Rheumatology in Europe collaborative group (2025) recently stressed the need to strengthen HL skills within the healthcare workforce [20], as assessing patients’ HL needs remains difficult [12,15].

In the present study setting, all HPs were trained in motivational interviewing and used a structured conversation template to support SDM. However, interpersonal variation in how these dialogues are conducted persists. Our previous interview study conducted in the same setting revealed that some patients felt poorly prepared and uncertain about what to expect from the goal-setting process, whereas others reported not feeling like ‘part of the team’ [9]. These findings highlight that elements of OHL, such as the quality of interpersonal communication, HPs’ attentiveness to patient needs, and the structural supports embedded within the organisation, may significantly influence patients’ SDM experiences [47]. To address these issues, a health-literate healthcare organisation is needed—1 in which HPs have a clear understanding of HL and access to practical tools for identifying and responding to HL needs. One such tool is the Conversational Health Literacy Assessment Tool (CHAT), which can help HPs explore 5 HL domains through structured dialogue [48]. Implementing tools like CHAT may strengthen OHL and support SDM across varying HL levels [47].

Individuals with low HL are often underrepresented in clinical studies due to barriers such as navigating written materials [11]. In our study, assistance with questionnaire completion—consistent with the Ophelia Manual (Optimising HL and Access) [49]—may have helped mitigate this issue. Interview-based data collection could further improve inclusion and is recommended for future studies. Although our sample spans the full HL spectrum, data from individuals with lower HL may be less precise due to lower representation. Although the sample is representative in terms of age and sex, participants reported higher educational levels than the broader patient population. Similar to the HL pattern, individuals with fewer educational resources appear underrepresented. Moreover, common biopsychosocial challenges among patients with RMDs, such as fatigue, pain, cognitive strain, or social stressors, may further burden individuals with lower HLQ scores across multiple scales, potentially affecting their ability to engage with study materials and thereby influencing study outcomes. Nonresponders were demographically similar to responders, reducing concern for systematic bias, though reasons for nonparticipation and potential differences should still be considered.

To measure patients’ perception of SDM, we chose the questionnaire CollaboRATE. Although this tool has been shown to be susceptible to ceiling effects [26,50], our use of the top score method revealed substantial response variability, with only 50% of participants rating SDM as optimal. This suggests that ceiling effects were not a dominant concern in our sample. Nonetheless, the top score approach may compress mid-range variation and underrepresent moderate but meaningful SDM experiences [50], which should be considered when interpreting the strength and shape of associations between HLQ scales and SDM. Although the individual HLQ subscales are well suited to identify whether interventions should address organisational or individual needs [28], our data do not explain the complexity of the observed relationships between HLQ scores and perceptions of SDM. As this was not a methodological study and no psychometric evaluation of the instruments was conducted, we can only speculate as to why certain HLQ scales showed linear versus nonlinear associations with SDM.

In this study, we applied a pragmatic classification into low, moderate, and high HLQ scores to support interpretability and pattern recognition. Although this approach offers a useful framework, it may not fully capture the multidimensional nature of HL, where individuals often demonstrate varied strengths across domains [25]. This might limit comparability across studies using alternative classification methods and supports the continued refinement of strategies for categorising HL skills.

### Conclusion

This study revealed a complex relationship between patients’ HL skills and their perceived involvement in SDM during goal setting in rehabilitation for patients with RMDs, as we found both linear and nonlinear relationships between different HL dimensions and SDM. The findings also call for clinical and organisational HL awareness of the diverse HL profiles among patients with RMDs. Tailored support from HPs appears important to facilitate meaningful SDM, particularly for individuals facing HL-related challenges. This may include those with moderate HL skills, who could be overlooked despite having expectations for involvement. Men also reported suboptimal SDM more frequently than women, highlighting the need to consider sex-related dynamics in person-centred care. Future research should explore how organisations and HPs can support patients with diverse HL skills to strengthen SDM in goal setting within rheumatology care. Further studies, including cluster analyses, are needed to clarify the mechanisms behind varying associations between HL dimensions and patient involvement in SDM.

## CRediT authorship contribution statement

**Lisbeth Skovly Nielsen:** Writing – original draft, Visualization, Validation, Resources, Project administration, Methodology, Investigation, Funding acquisition, Formal analysis, Data curation, Conceptualization. **Kirsten Lykke Knak:** Writing – original draft, Validation, Supervision, Project administration, Methodology, Conceptualization. **Brian Clausen:** Writing – original draft, Validation, Supervision, Project administration, Methodology, Conceptualization. **Kamila Adellund Holt:** Writing – original draft, Validation, Supervision, Methodology, Conceptualization. **Sofie Ronja Petersen:** Writing – original draft, Visualization, Validation, Supervision, Software, Methodology, Investigation, Formal analysis, Data curation. **Jette Primdahl:** Writing – original draft, Validation, Supervision, Resources, Project administration, Methodology, Investigation, Funding acquisition, Conceptualization. **Ann Bremander:** Writing – original draft, Validation, Supervision, Resources, Project administration, Methodology, Investigation, Funding acquisition, Data curation, Conceptualization.

## Competing interests

All authors declare they have no competing interests.

## Appendix Supplementary materials

Image, application 1

Image, application 2

Image, application 3
