# Supplementary material for: Unravelling complex relationships between health literacy and shared decision-making: a cross-sectional study in patients attending rheumatology rehabilitation
Source: EULAR Rheumatol Open. 2026 Mar 5;2(1):314–22. doi: 10.1016/j.ero.2026.02.014 (PMC13292362; doi:10.1016/j.ero.2026.02.014)
Supplement: Supplementary file 1 [file mmc1.docx]

# Checklist for Reporting Results of Internet E‑Surveys (CHERRIES)

*“Unravelling complex relationships between Health Literacy and Shared Decision Making: A cross-sectional study in patients attending rheumatology rehabilitation“* (Submission ID ERO-D-25-00206).

| **Item Category** | **Checklist Item** | **Answer** |
| --- | --- | --- |
| **Design** | Describe survey design | Cross-sectional closed survey (eligible participants only) via email/personal link; identifiable via coded ID. |
| **Ethics & Consent** | Ethics approval | In Denmark, survey studies do not require formal approval from regional ethics committees  (case no: 20202000-117). GDPR (general data protection act) restricts use and distribution of data. |
|  | Informed consent | Participants received oral and written information prior to giving their written consent. |
|  | Data protection | Data were securely stored within the OPEN platform, a safe environment that comply with the European General Data Protection Regulations and the Danish law for data security. |
| **Development & Pre‑testing** | Development and testing | The survey used validated questionnaires and was pilot‑tested among patient representatives and clinicians. |
| **Recruitment** | Open vs. closed survey | Closed survey (password/personal link distribution to invited participants). |
|  | Contact mode and advertising | Participants were invited at an initial consultation prior to a rehabilitation admission and received a personal link after consent (sent by email or handed out on paper if preferred). No advertising. |
| **Survey Administration** | Web/e-mail | Research Electronic Data Capture (REDCap). Access via a personal link, completed on computer, phone, or tablet. |
|  | Context | In-patient rehabilitation |
|  | Mandatory/voluntary | Voluntary |
|  | Incentives | No incentives were offered |
|  | Time/date | April 2021 – June 2022. |
|  | Randomization of questionnaires | NA |
|  | Adaptive questioning | Applied throughout the survey where relevant. |
|  | Number of items/pages | 80 items, 12 pages. |
|  | Completeness check | The participants could not proceed in the survey until they had answered the question. Response options included “not applicable” and “other” with a text field provided, where relevant. |
|  | Review step | The questionnaire had a final page with a summary of responses. The participants had the opportunity to go back and change answers. |
|  | Unique site visitor | Multiple submissions/entries were restricted (system controls) to avoid duplicates. |
|  | View rate | NA |
| **Response & Participation** | Participation rate | N= 514 |
|  | Completion rate | 408 (28% of these were paper‑based responses subsequently entered manually in REDCap); completed: 401; incomplete by a few items: 7. |
| **Preventing multiple entries** | Cookies used, IP, log file analysis | A unique user identifier based on a personal identification number was used to prevent duplicates. On a second attempt to log in after the survey was finished, a note with “thank you for your participation” appeared. |
| **Handling Incomplete Data** | Inclusion/exclusion of partials | Only complete responses for CollaboRATE were analysed. Missing data from the independent variables were accounted for by the maximum likelihood approach in the primary model. |
|  | Questionnaires submitted with an atypical timestamp | NA |
|  | Statistical correction, weighing of items to adjust for non-representative sample. | Missing data from the independent variables were accounted for by the maximum likelihood approach in the primary model. |
