# Supplementary material for: Unravelling complex relationships between health literacy and shared decision-making: a cross-sectional study in patients attending rheumatology rehabilitation
Source: EULAR Rheumatol Open. 2026 Mar 5;2(1):314–22. doi: 10.1016/j.ero.2026.02.014 (PMC13292362; doi:10.1016/j.ero.2026.02.014)
Supplement: Supplementary file 2 [file mmc2.pdf]

Spørgeskemaundersøgelse

Sundhedskompetence og fælles beslutningstagen

Tak fordi du har sagt ja til at deltage i denne spørgeskemaundersøgelse.

Vi vil gerne undersøge hvordan og i hvilket omfang du oplever at deltage i de beslutninger, der handler om mål og ønsker for dit ophold. Derudover ønsker vi at undersøge din oplevelse af at færdes i sundhedsvæsnet.

Det tager dig ca. **25 minutter** at besvare de følgende spørgsmål.

Når du har udfyldt spørgeskemaerne, skal du aflevere dem **i postkassen ved receptionen på sengeafdelingen**.

ID: _______

Udfyldes af projektmedarbejder

Dansk Gigthospital

Sano

De første spørgsmål omhandler din baggrund

Angiv dags dato

____

Dato – måned – år

Hvad er dit CPR-nummer?

Eksempel:

1	1	0	1	5	1	-	1	4	1	7

Angiv dit CPR-nummer:

						-				

Hvilken dato blev du indlagt til rehabilitering på Dansk Gigthospital?

____

Dato – måned – år

Hvilken sygdom er grunden til, at du skal indlægges til rehabilitering?

Hvis du har flere sygdomme, bedes du sætte **ét** kryds ved din primære diagnose.

☐ Leddegigt (reumatoid artrit)

☐ Slidgigt (artrose)

☐ Rygsøjlegigt (Mb. Bechterew)

☐ Muskelgigt

☐ Knogleskørhed (osteoporose)

☐ Psoriasis-gigt (psoriasis artrit)

☐ Urinsyregigt

☐ Sjögrens syndrom

☐ Fibromyalgi

(fortsættes på næste side)

☐ Lupus

☐ Hypermobilitet

☐ Discusprolaps

☐ Ved ikke

☐ Anden diagnose, angiv hvilken: ____

Hvornår startede dine symptomer?

____

Angiv ca. årstal, fx "1998"

Hvornår fik du denne diagnose af en læge?

____

Angiv ca. årstal, fx "2010"

Har du – ud over denne diagnose - andre helbredsproblemer?

Sæt gerne flere krydser

☐ Forhøjet blodtryk

☐ Sukkersyge (diabetes type 1 eller 2)

☐ Hjertekramper (angina pectoris)

☐ Blodprop i hjertet

☐ Hjerneblødning/blodprop i hjernen

☐ Forhøjet kolesteroltal

☐ Åreforkalkning i benene

☐ Anden hjerte-kar-sygdom

☐ Kronisk bronkitis

☐ KOL (Kronisk Obstruktiv Lungelidelse)

☐ Astma

(fortsættes på næste side)

☐ Psoriasis

☐ Knogleskørhed (osteoporose)

☐ Stofskiftesygdom

☐ Mavesår

☐ Leverlidelse

☐ Nyrelidelse

☐ Dissemineret sklerose

☐ Depression

☐ Psykisk lidelse (som har varet mere end 6 måneder)

☐ Inflammatorisk tarmsygdom

☐ Kræft

☐ Slidgigt (artrose)

☐ Leddegigt (reumatoid artrit)

☐ Discusprolaps eller andre rygsygdomme

☐ Migræne eller hyppig hovedpine

☐ Anden diagnose, angiv hvilken: ____________________________________

☐ Nej – jeg har ingen andre helbredsproblemer

Hvilken skole- eller ungdomsuddannelse har du?

Sæt ét kryds

☐ 7 eller færre års skolegang

☐ 8 til 9 års skolegang

☐ 10 til 11 års skolegang

☐ Gymnasial uddannelse, studenter- eller HF-eksamen (herunder HHX og HTX)

Har du fuldført en uddannelse ud over skole- eller ungdomsuddannelse?

☐ Ja

☐ Nej

☐ Jeg er under uddannelse

Hvis du har svaret ja, hvilken uddannelse har du da fuldført?

☐ Ét eller flere kortere kurser
(f.eks. specialarbejderkurser, arbejdsmarkedskurser mv.)

☐ Erhvervsfaglig uddannelse /faglært
(f.eks. kontor- eller butiksassistent, frisør, murer, social- og sundhedsassistent mv.)

☐ Kort videregående uddannelse (2-3 år)
(f.eks. finansøkonom, multimediedesigner, tandplejer, laborant mv.)

☐ Mellemlang videregående uddannelse (3-4 år)
(f.eks. lærer, bygningskonstruktør, sygeplejerske, diplomingeniør, pædagog mv.)

☐ Lang videregående uddannelse (over 4 år)
(f.eks. civilingeniør, cand.mag., læge, psykolog mv.)

☐ Anden uddannelse

Har du tidligere været indlagt til rehabilitering på enten Dansk Gigthospital eller Sano?

☐ Ja

☐ Nej

Hvis **ja**, i hvilket år var du da senest indlagt til rehabilitering (ca.) _________

De kommende spørgsmål handler om dine ønsker og mål for dit rehabiliteringsophold. Vi spørger også til din oplevelse af at være inddraget i beslutninger omhandlende dit ophold.

Kan du huske, hvad du og dit team af behandlere (fysio- og ergoterapeut, sygeplejerske og læge) har besluttet, at ønskerne/målene for dit rehabiliteringsophold skal være?

☐ Ja

☐ Nej

Hvis ja, beskriv da med egne ord dine ønsker/mål for dit rehabiliteringsophold

Mine ønsker/mål:

Eventuelle øvrige kommentarer:

Tænk tilbage på de samtaler du har haft med dine behandlere i løbet af den første tid af dit ophold.

Sæt ring om det tal på vurderingsskalaen, der passer bedst til din oplevelse.

I hvor høj grad blev der gjort en indsats for at hjælpe dig med at forstå din helbredssituation?

Slet ikke **I høj grad**

**0**	**1**	**2**	**3**	**4**	**5**	**6**	**7**	**8**	**9**

I hvor høj grad blev der gjort en indsats for at høre, hvad der betyder mest for dig, når det gælder din helbredssituation?

Slet ikke **I høj grad**

**0**	**1**	**2**	**3**	**4**	**5**	**6**	**7**	**8**	**9**

I hvor høj grad blev der gjort en indsats for at inddrage det, der er vigtigt for dig, i beslutningen om, hvad der nu skal ske?

Slet ikke **I høj grad**

**0**	**1**	**2**	**3**	**4**	**5**	**6**	**7**	**8**	**9**

Eventuelle kommentarer:

Spørgeskema om sundhed og sundhedsvæsen

I dette spørgeskema stiller vi spørgsmål om, hvordan du finder og bruger sundhedsinformation, og om hvordan du tager dig af din sundhed og samarbejder med læger og andre sundhedsprofessionelle.

I spørgeskemaet bruger vi ordet **sundhedsprofessionelle**. Hermed mener vi praktiserende læger, andre læger, sygeplejersker, fysioterapeuter, diætister og andre professionelle, som du møder i sundhedsvæsenet, eller som du får råd fra om sundhed.

Vi bruger ordet **sundhedsydelse** om fx behandling, pleje, træning eller anden kontakt, du har med sundhedsprofessionelle.

The Health Literacy Questionnaire (HLQ). © Copyright 2014 Swinburne University of Technology.
Authors: Richard Osborne, Rachelle Buchbinder, Roy Batterham, Gerald Elsworth.
No part of the HLQ can be reproduced, copied, altered or translated without the permission of the authors.
Further information: ghe-licences@swin.edu.au

Information om spørgeskemaet og hvordan det skal udfyldes

Spørgeskemaet har to dele.

I **del 1** bliver du bedt om at besvare **hvor uenig eller enig**, du er i et udsagn.
I **del 2** bliver du bedt om at besvare **hvor svært eller let**, du finder forskellige opgaver.
For hvert udsagn eller opgave skal du sætte kryds, ved det der beskriver dig lige nu.

Tjek venligst, at du sætter kryds ud for alle spørgsmål.

Et eksempel:

	Meget uenig	Uenig	Enig	Meget enig
1. Jorden er flad	☒	☐	☐	☐

Jane Nielsen har besvaret, at hun er **meget uenig** i, at jorden er flad

Første del af spørgeskemaet starter her

Angiv hvor **uenig** eller **enig** du er i nedenstående udsagn.

Sæt kun et kryds ved hvert udsagn.

Tjek venligst, at krydset er sat i boksen: ☒

	Meget uenig	Uenig	Enig	Meget enig
1. Jeg føler mig godt informeret om sundhed	☐	☐	☐	☐
2. Der er mindst én sundhedsprofessionel, som kender mig godt	☐	☐	☐	☐
3. Jeg har mange, jeg kan kontakte, der forstår mig og støtter mig	☐	☐	☐	☐
4. Jeg sammenligner informationer om sundhed fra forskellige steder	☐	☐	☐	☐
5. Når jeg har det dårligt, har mine omgivelser virkelig forståelse for, hvordan jeg har det	☐	☐	☐	☐
6. Jeg bruger en hel del tid på at tage mig af mit helbred og min sundhed	☐	☐	☐	☐
7. Når jeg støder på nye informationer om sundhed, undersøger jeg, om de er rigtige	☐	☐	☐	☐

Angiv hvor **uenig** eller **enig** du er i nedenstående udsagn.

Sæt kun ét kryds ved hvert udsagn.

	Meget uenig	Uenig	Enig	Meget enig
8. Der er mindst én sundhedsprofessionel, jeg kan tale med om mine sundhedsproblemer	☐	☐	☐	☐
9. Jeg planlægger, hvordan jeg holder mig sund og rask	☐	☐	☐	☐
10. Jeg har nok information til, at jeg kan tage mig af mine helbredsproblemer	☐	☐	☐	☐
11. Hvis jeg har brug for hjælp, har jeg mange mennesker, jeg kan regne med	☐	☐	☐	☐
12. Jeg sammenligner altid informationer om sundhed fra forskellige steder, før jeg beslutter, hvad der er bedst for mig	☐	☐	☐	☐
13. Jeg tager mig tid til at leve sundt, uanset hvad der ellers sker i mit liv	☐	☐	☐	☐
14. Jeg er sikker på, at jeg har nok information til at tage mig bedst muligt af mit helbred	☐	☐	☐	☐
15. Jeg har mindst én person, der kan tage med mig til lægen	☐	☐	☐	☐
16. Jeg ved, hvordan jeg finder ud af, om den information jeg får om sundhed, er rigtig	☐	☐	☐	☐
17. Jeg har adgang til sundhedsprofessionelle, der kan hjælpe mig med at finde ud af, hvad jeg skal gøre	☐	☐	☐	☐
18. Jeg sætter mine egne mål for min sundhed	☐	☐	☐	☐
19. Jeg har stor støtte fra familie og venner	☐	☐	☐	☐
20. Jeg spørger sundhedsprofessionelle om, hvor god den information er, jeg selv har fundet	☐	☐	☐	☐
21. Der er ting, jeg gør regelmæssigt for at blive sundere	☐	☐	☐	☐
22. Der er mindst én sundhedsprofessionel, jeg kan regne med	☐	☐	☐	☐
23. Jeg har al den information, jeg behøver for at passe på mit helbred	☐	☐	☐	☐

Anden del af spørgeskemaet starter her

Angiv hvor **svært** eller **let** du finder nedenstående opgaver lige nu.

Sæt kun ét kryds ved hvert udsagn.

Tjek venligst, at krydset er sat i boksen: ☒

	Kan ikke eller altid svært	Oftest svært	Nogle gange svært	Oftest let	Altid let
1. Finde de rigtige tilbud i sundhedsvæsnet	☐	☐	☐	☐	☐
2. Sikre dig, at sundhedsprofessionelle forstår dine problemer rigtigt	☐	☐	☐	☐	☐
3. Finde informationer om helbredsproblemer	☐	☐	☐	☐	☐
4. Være i stand til at tale om dine helbredsproblemer med en sundhedsprofessionel	☐	☐	☐	☐	☐
5. Udfylde skemaer med sundhedsoplysninger rigtigt	☐	☐	☐	☐	☐
6. Finde information om sundhed fra forskellige steder	☐	☐	☐	☐	☐
7. Have gode samtaler om dit helbred med din læge	☐	☐	☐	☐	☐
8. Få en tid hos de sundhedsprofessionelle, du har brug for	☐	☐	☐	☐	☐
9. Præcist følge de anvisninger, du får af sundhedsprofessionelle	☐	☐	☐	☐	☐
10. Få relevant information om sundhed, så du er godt opdateret	☐	☐	☐	☐	☐
11. Afgøre, hvilken type sundhedsprofessionel, du har brug for at opsøge	☐	☐	☐	☐	☐
12. Læse og forstå skriftlig information om sundhed	☐	☐	☐	☐	☐
13. Finde frem til det rigtige sted at få de sundhedsydelser, du har brug for	☐	☐	☐	☐	☐
14. Få information om sundhed i et forståeligt sprog	☐	☐	☐	☐	☐
15. Blive ved med at spørge den sundhedsprofessionelle, indtil du har forstået det, du har brug for	☐	☐	☐	☐	☐

Anden del fortsætter her

Angiv hvor **svært** eller **let** du finder nedenstående opgaver lige nu.

Sæt kun ét kryds ved hvert udsagn.

	Kan ikke eller altid svært	Oftest svært	Nogle gange svært	Oftest let	Altid let
16. Finde ud af, hvilke sundhedsydelser du har ret til	☐	☐	☐	☐	☐
17. Læse og forstå al information på medicinpakninger	☐	☐	☐	☐	☐
18. Selv finde informationer om sundhed	☐	☐	☐	☐	☐
19. Finde frem til, hvad der er den bedste sundhedsydelse for dig	☐	☐	☐	☐	☐
20. Stille spørgsmål til sundhedsprofessionelle for at få den information, du har brug for	☐	☐	☐	☐	☐
21. Forstå, hvad sundhedsprofessionelle vil have dig til at gøre	☐	☐	☐	☐	☐

Eventuelle kommentarer

Nedenfor er en liste over symptomer eller problemer, man kan have. Du bedes vurdere, hvor meget du har følt dig generet eller ulykkelig over dem den sidste uges tid, inklusiv i dag.

Sæt et kryds ud for det svar, der passer bedst på dig.

Føler dig angst

☐ Slet ikke

☐ Lidt

☐ Meget

☐ Ekstremt meget

Fornemmelse af nervøsitet eller indvendig rysten

☐ Slet ikke

☐ Lidt

☐ Meget

☐ Ekstremt meget

Ingen tro på fremtiden

☐ Slet ikke

☐ Lidt

☐ Meget

☐ Ekstremt meget

Tristhedsfølelse

☐ Slet ikke

☐ Lidt

☐ Meget

☐ Ekstremt meget

Er overbekymret

☐ Slet ikke

☐ Lidt

☐ Meget

☐ Ekstremt meget

EQ-5D-5L

Helbredsspørgeskema

Dansk version for Danmark

(Danish version for Denmark)

Denmark (Danish) © 2009 EuroQol Group EQ-5D™ is a trade mark of the EuroQol Group

Under hver overskrift bedes du sætte kryds i DEN kasse, der bedst beskriver dit helbred I DAG.

BEVÆGELIGHED

Jeg har ingen problemer med at gå omkring ❑

Jeg har lidt problemer med at gå omkring ❑

Jeg har moderate problemer med at gå omkring ❑

Jeg har store problemer med at gå omkring ❑

Jeg kan ikke gå omkring ❑

PERSONLIG PLEJE

Jeg har ingen problemer med at vaske mig eller klæde mig på ❑

Jeg har lidt problemer med at vaske mig eller klæde mig på ❑

Jeg har moderate problemer med at vaske mig eller klæde mig på ❑

Jeg har store problemer med at vaske mig eller klæde mig på ❑

Jeg kan ikke vaske mig eller klæde mig på ❑

SÆDVANLIGE AKTIVITETER *(fx. arbejde, studie, husarbejde, familie- eller fritid)*

Jeg har ingen problemer med at udføre mine sædvanlige aktiviteter ❑

Jeg har lidt problemer med at udføre mine sædvanlige aktiviteter ❑

Jeg har moderate problemer med at udføre mine sædvanlige aktiviteter ❑

Jeg har store problemer med at udføre mine sædvanlige aktiviteter ❑

Jeg kan ikke udføre mine sædvanlige aktiviteter ❑

SMERTER / UBEHAG

Jeg har ingen smerter eller ubehag ❑

Jeg har lidt smerter eller ubehag ❑

Jeg har moderate smerter eller ubehag ❑

Jeg har stærke smerter eller ubehag ❑

Jeg har ekstreme smerter eller ubehag ❑

ANGST / DEPRESSION

Jeg er ikke ængstelig eller deprimeret ❑

Jeg er lidt ængstelig eller deprimeret ❑

Jeg er moderat ængstelig eller deprimeret ❑

Jeg er meget ængstelig eller deprimeret ❑

Jeg er ekstremt ængstelig eller deprimeret ❑

- Vi vil gerne vide, hvor godt eller dårligt dit helbred er I DAG.
- Denne skala er nummereret fra 0 til 100.
- 100 svarer til det <u>bedste</u> helbred, du kan forestille dig.
  0 svarer til det <u>dårligste</u> helbred, du kan forestille dig.
- Sæt et X på det sted på skalaen, der viser, hvordan dit helbred er I DAG.
- Skriv derefter det tal, du har markeret på skalaen, ind i boksen nedenunder.

Det bedste helbred, du kan forestille dig

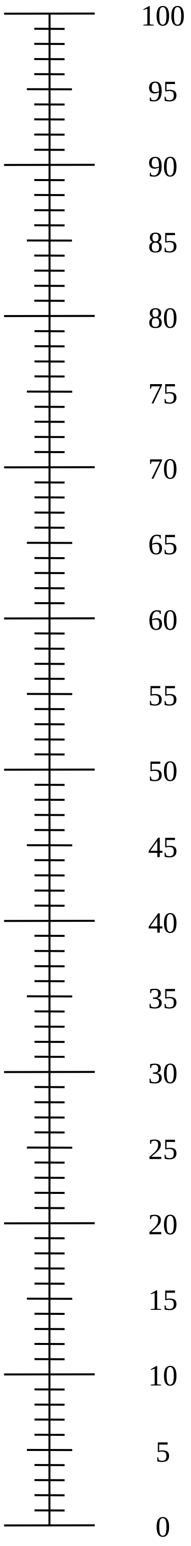

Det dårligste helbred, du kan forestille dig

DIT HELBRED I DAG = ☐

Tusind tak for din hjælp!

Du er nu færdig med at besvare spørgeskemaerne, og du bedes aflevere dem **i postkassen ved receptionen på sengeafdelingen**.
