# Supplementary material for: Unravelling complex relationships between health literacy and shared decision-making: a cross-sectional study in patients attending rheumatology rehabilitation
Source: EULAR Rheumatol Open. 2026 Mar 5;2(1):314–22. doi: 10.1016/j.ero.2026.02.014 (PMC13292362; doi:10.1016/j.ero.2026.02.014)
Supplement: Supplementary file 3 [file mmc3.docx]

# Survey on Health Literacy and Shared Decision-Making (English Review Version)

*This English translation is provided for review purposes only and is not a validated version of the instrument. HLQ items are omitted due to licensing restrictions.*

## Introduction

Thank you for agreeing to participate in this survey study. We would like to examine how and to what extent you feel involved in decisions regarding the goals and expectations for your stay. We would also like to understand your experience navigating the healthcare system.

It will take approximately 25 minutes to complete the following questions. When you have finished completing the questionnaires, please return them to the mailbox located at the ward reception.

## Background Questions

- Please indicate today’s date (day–month–year): ___________________________

- What is your CPR number? *Example boxes shown in original Danish version.*

- Date of admission for rehabilitation at the Danish Hospital for Rheumatic Diseases (day–month–year): ___________________________

- What is the primary diagnosis for which you are admitted to rehabilitation? (Select one). *Listing of diagnoses (full list available in the original Danish version)*

- When did your symptoms begin? Please state approximate year (e.g., “1998”).

- When were you diagnosed by a physician? Please state approximate year (e.g., “2010”).

- Do you have any other health problems besides your primary diagnosis? (Select all that apply). *Listing of diagnoses (full list available in the original Danish version)*

- What is your highest completed level of school or youth education? *Listing of education levels provided in the original Danish version)*

- Have you completed any education beyond school or youth education?

- If yes, which type of education have you completed?

- Have you previously been admitted for rehabilitation at either the Danish Hospital for Rheumatic Diseases or Sano?

## Goals and Preferences for Your Rehabilitation Stay

Can you remember what you and your treatment team (physiotherapists, occupational therapists, nurses, and physician) agreed as the goals for your rehabilitation stay? (Yes/No)

If yes, please describe your goals in your own words: ____________________________________________

## Shared Decision-Making Items (CollaboRATE)

Think back to the conversations you have had with your healthcare providers during the first part of your stay. Circle the number that best reflects your experience. Scale from 0 (Not at all) to 9 (A great deal).

- How much effort was made to help you understand your health situation? (0–9 scale)

- How much effort was made to listen to what matters most to you regarding your health? (0–9 scale)

- How much effort was made to include what matters most to you in deciding what to do next? (0–9 scale)

## HLQ Section (Omitted)

HLQ items are omitted due to licensing restrictions.

## Symptom Questionnaire

Below is a list of symptoms or problems a person may experience. Please assess how much that have bothered or distressed you during the past week, including today. Mark the answer that best applies to you.

- Feeling anxious: Not at all / A little / A lot / Extremely

- Feeling nervous or internally shaky: Not at all / A little / A lot / Extremely

- Lack of faith in the future: Not at all / A little / A lot / Extremely

- Feeling sad: Not at all / A little / A lot / Extremely

- Feeling overly worried: Not at all / A little / A lot / Extremely

## EQ-5D (Standard English Wording)

Under each heading, please indicate the statement that best describes your health TODAY.

### Mobility

- I have no problems walking about

- I have slight problems walking about

- I have moderate problems walking about

- I have severe problems walking about

- I am unable to walk about

### Self-care

- I have no problems washing or dressing myself

- I have slight problems washing or dressing myself

- I have moderate problems washing or dressing myself

- I have severe problems washing or dressing myself

- I am unable to wash or dress myself

### Usual activities

- I have no problems doing my usual activities

- I have slight problems doing my usual activities

- I have moderate problems doing my usual activities

- I have severe problems doing my usual activities

- I am unable to do my usual activities

### Pain/Discomfort

- I have no pain or discomfort

- I have slight pain or discomfort

- I have moderate pain or discomfort

- I have severe pain or discomfort

- I have extreme pain or discomfort

### Anxiety/Depression

- I am not anxious or depressed

- I am slightly anxious or depressed

- I am moderately anxious or depressed

- I am severely anxious or depressed

- I am extremely anxious or depressed

### EQ Visual Analogue Scale

Please indicate how good or bad your health is TODAY, on a scale from 0 to 100 (0 = worst health you can imagine, 100 = best health you can imagine).

Your health today = ______

Thank you very much for your help! When finished, please return the completed questionnaire to the mailbox at the ward reception.
